# Supplementary material for: Multiple myeloma risk variant at 7p15.3 creates an IRF4-binding site and interferes with CDCA7L expression
Source: Nat Commun. 2016 Nov 24;7:13656. doi: 10.1038/ncomms13656 (PMC5123067; doi:10.1038/ncomms13656)
Supplement: Supplementary Information — Supplementary Figures 1-8, Supplementary Tables 1-4 and Supplementary References [file ncomms13656-s1.pdf]

Supplementary Figure 1. eQTL boxplots.

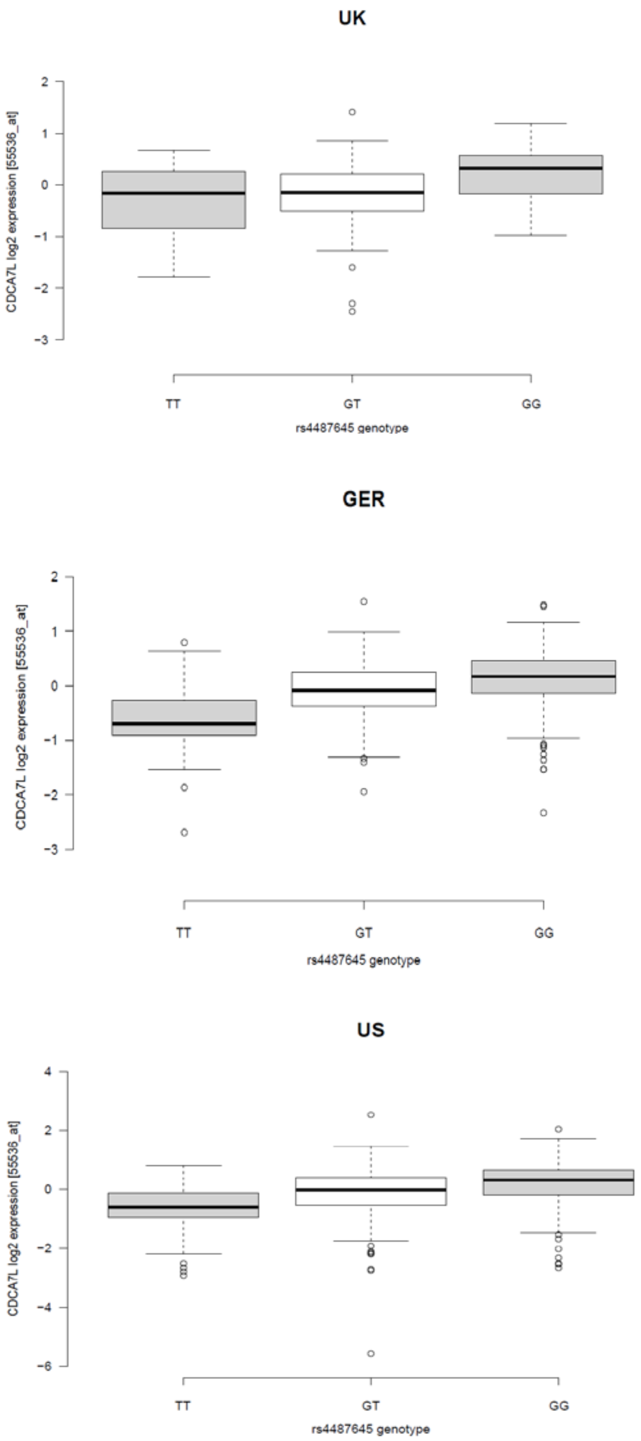

Meta *P* = 1.95×10^{-36}

Relationship between rs4487645 genotype and *CDCA7L* expression in CD138+ selected plasma cells from 658 German (GER) (E-MTAB-2299), 183 UK (GSE21349) and 608 US (GSE2658, GSE31161) MM patients. The central line in each box indicates the median; the bottom and top lines of the box are the 25th and 75th percentiles; whiskers extend 1.5 times from the 25th and 75thpercentiles.

Supplementary Figure 2. Expression correlation between *IRF4* and *CDCA7L* in MM.

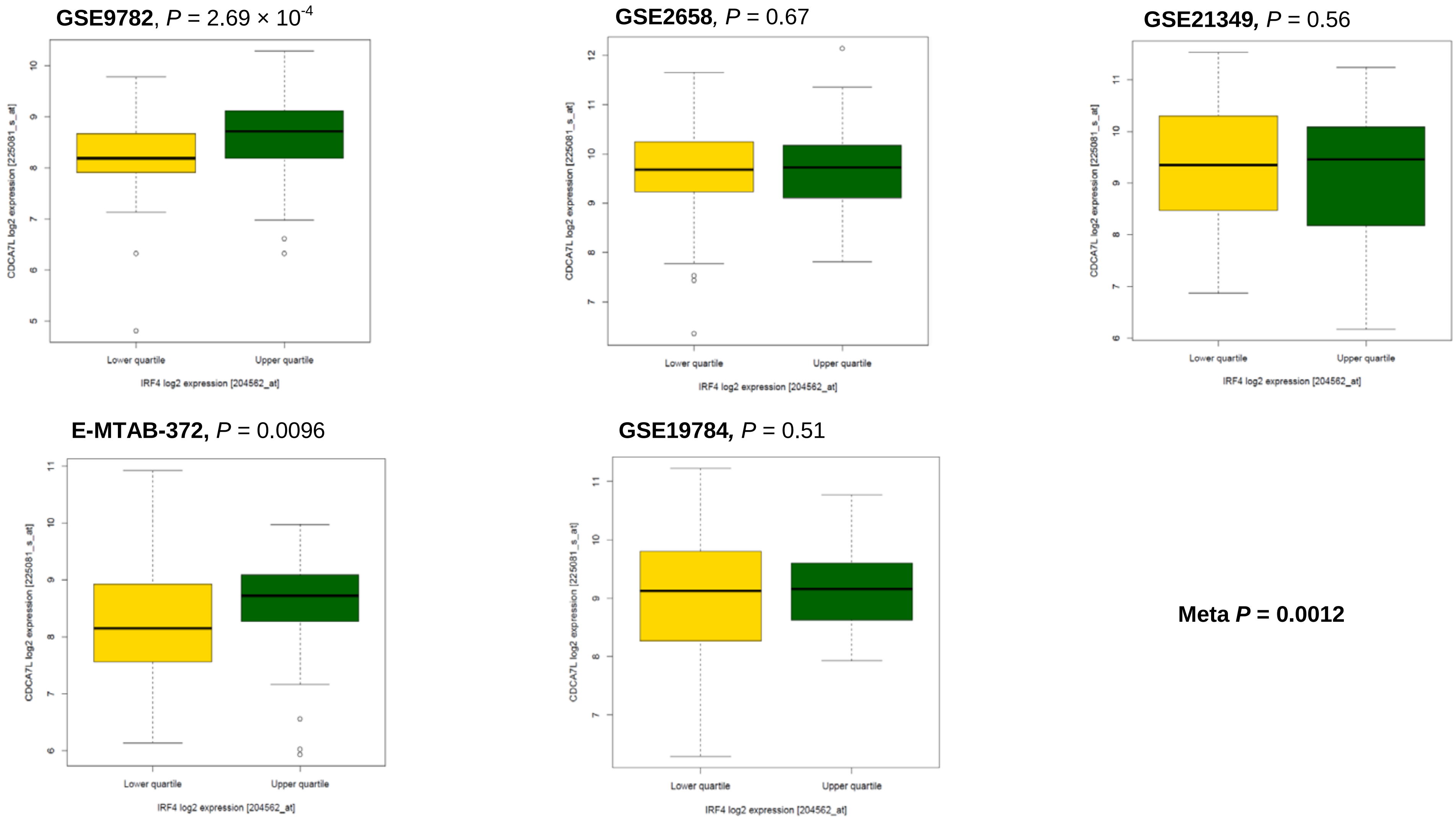

Relationship between differential *IRF4* expression (upper and lower quartiles) and *CDCA7L* expression in multiple myeloma expression datasets GSE9782, GSE2658, GSE19784, E-MTAB-372 and GSE21349. The central line in each box indicates the median; the bottom and top lines of the box are the 25th and 75th percentiles; whiskers extend 1.5 times from the 25th and 75th percentiles. Statistical analysis was performed with Mann-Whitney-Wilcoxon test. *P* values were combined from independent datasets combined using Fisher's Method.

Supplementary Figure 3. *IRF4* knockdown reduces expression of *CDCA7L*.

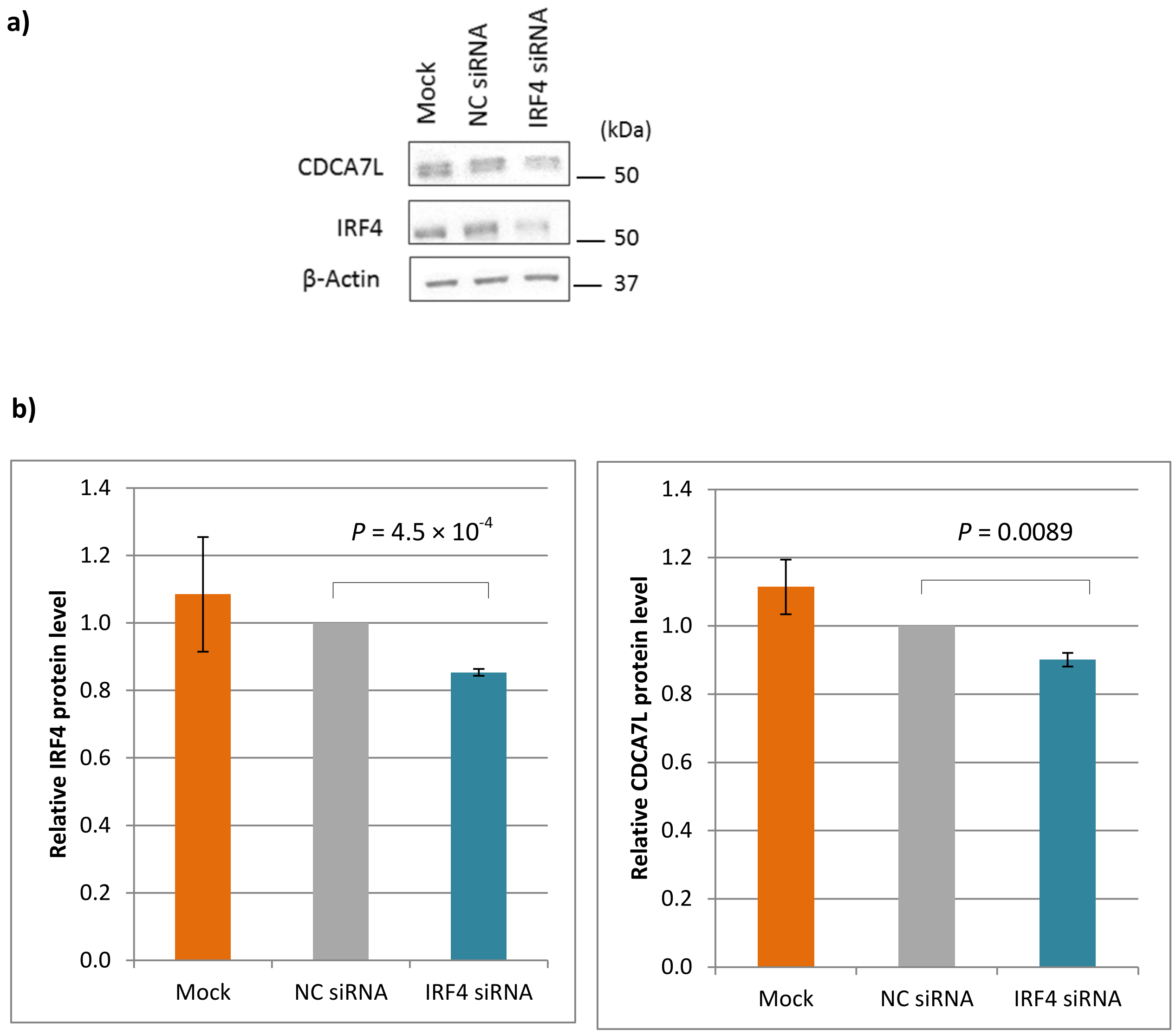

a) Representative Western blot analysis of IRF4 and CDCA7L protein level in KMS11 cells 24 h post-transfection with indicated siRNAs. **b)** Quantification of protein levels with ImageJ software in mock transfection and *IRF4* knockdown normalized to NC siRNA. Data shown are mean ± SEM from three biological experiments and assessed by two-tailed *t*-test. The protein samples from the three biological replicates were derived and processed as one experiment, and the blots were generated and processed in parallel.

Supplementary Figure 4. Expression correlation between *MYC* and *CDCA7L* in MM.

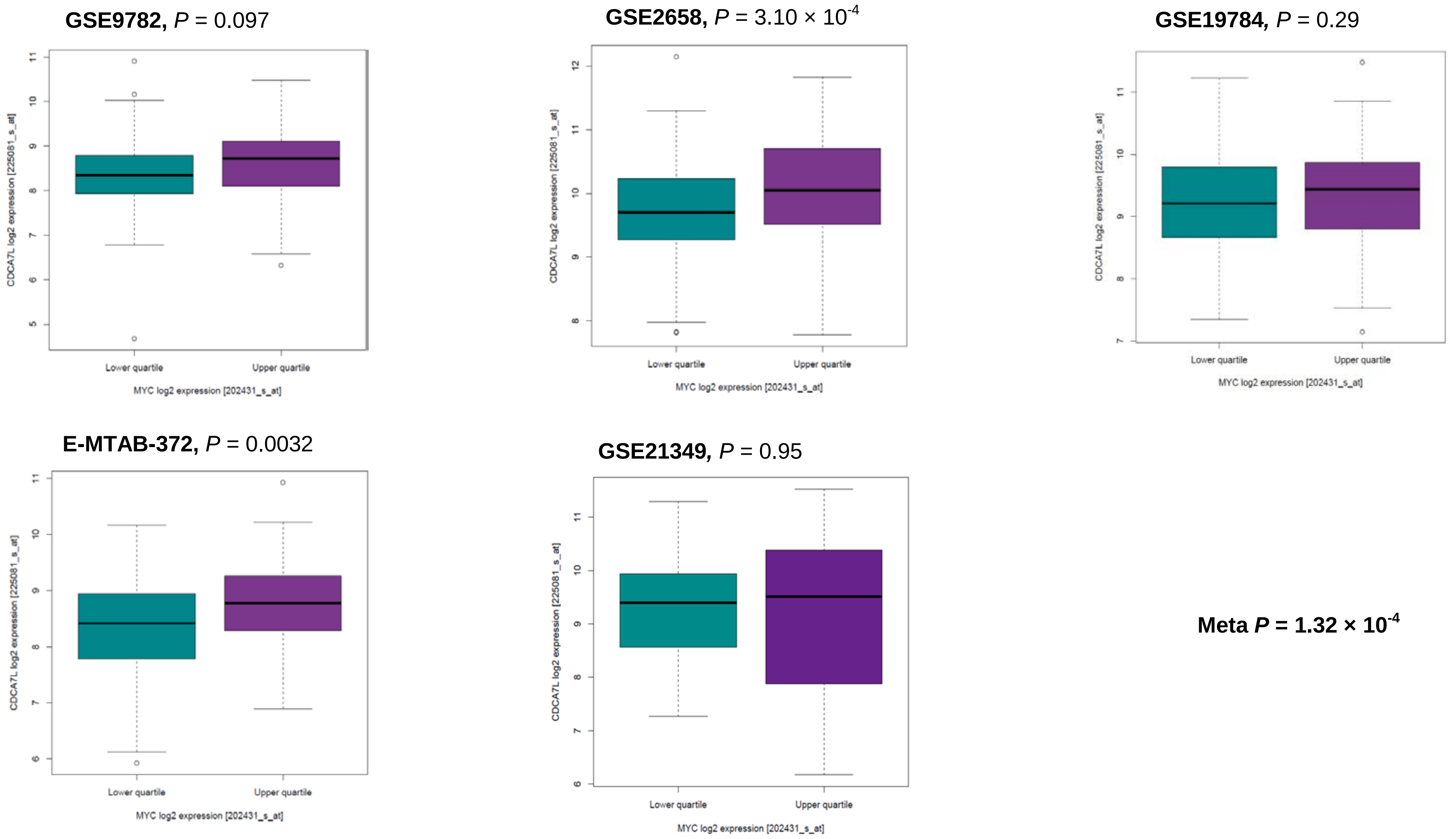

Relationship between differential *MYC* expression (upper and lower quartiles) and *CDCA7L* expression in multiple myeloma expression datasets GSE9782, GSE2658, GSE19784, E-MTAB-372 and GSE21349. The central line in each box indicates the median; the bottom and top lines of the box are the 25th and 75th percentiles; whiskers extend 1.5 times from the 25th and 75th percentiles. Statistical analysis performed with Mann-Whitney-Wilcoxon test. *P* values from independent datasets were combined using Fisher's Method.

Supplementary Figure 5. Relationship between a gene expression-based proliferation index and *CDCA7L* in MM patients.

a) GER

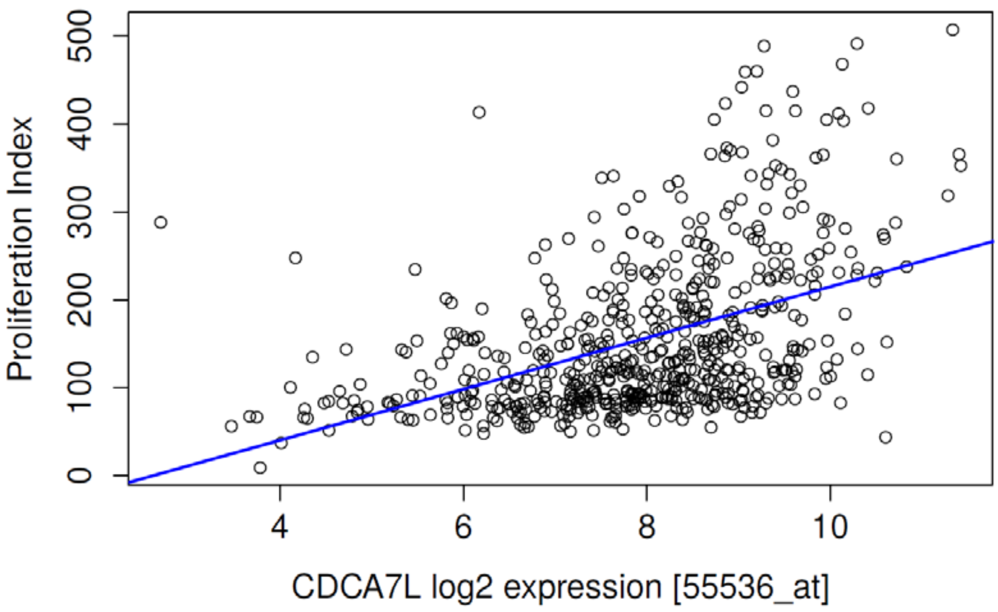

Pearson's product-moment correlation = 0.44, $P < 1.0 \times 10^{-16}$

b) US

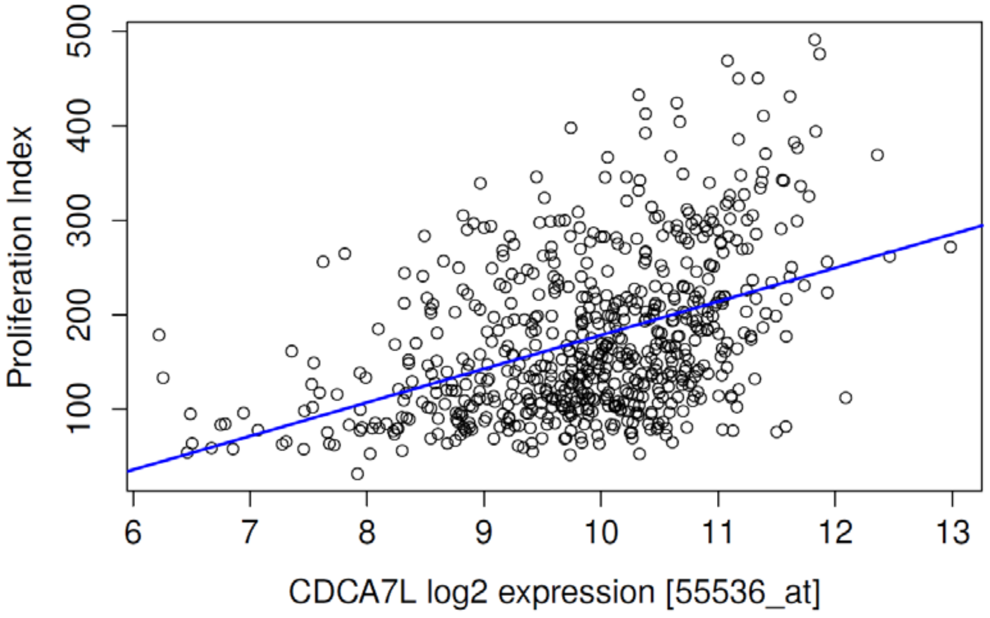

Pearson's product-moment correlation = 0.47, $P < 1.0 \times 10^{-16}$

Correlation plot of *CDCA7L* expression and gene expression-based proliferation index (GPI)[1] of GC-RMA (robust multiarray averaging) normalized Affymetrix U133+2 array expression from CD138+ selected MM plasma cells from **a)** 658 German MM patients (E-MTAB-2299) and **b)** 608 US MM patients (GSE2658, GSE31161). Blue line represents a line of best fit.

Supplementary Figure 6. *CDCA7L* knockdown in KMS11.

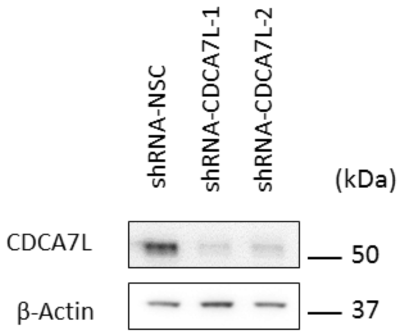

The knockdown efficiencies of shRNA-CDCA7L-1 and shRNA-CDCA7L-2 in KMS11 were determined by Western blotting 72 h after doxycycline was added to induce knockdown. β-actin was used as loading control. Representative Western blot shown below.

Supplementary Figure 7. Impact of *CDCA7L* knockdown on cell cycle.

a)

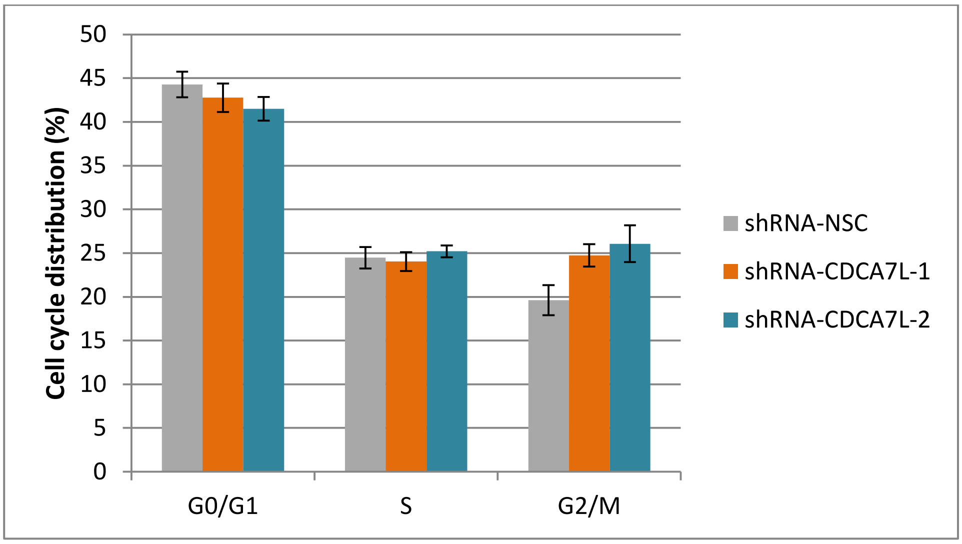

b)

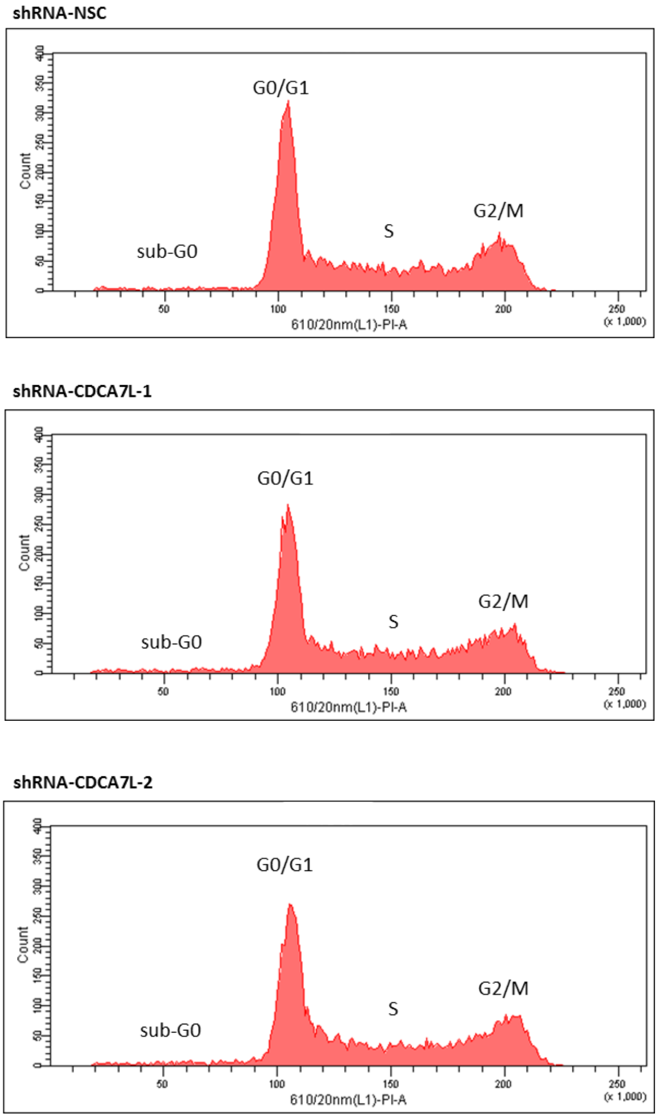

a) Cell cycle analysis by flow cytometry in transduced KMS11 with shRNA-CDCA7L-1, shRNA-CDCA7L-2 and shRNA-NSC 72 h after addition of doxycycline. Data shown are mean ± SEM from three independent experiments performed in triplicates. Differences at each cell cycle stage (%) between *CDCA7L* and control knockdowns were assessed by two-tailed *t*-test. **b)** Representative panels of cell cycle distribution between shRNA-CDCA7L-1, shRNA-CDCA7L-2 and shRNA-NSC at 72 h.

Supplementary Figure 8. Relationship between differential *CDCA7L* expression and patient overall survival.

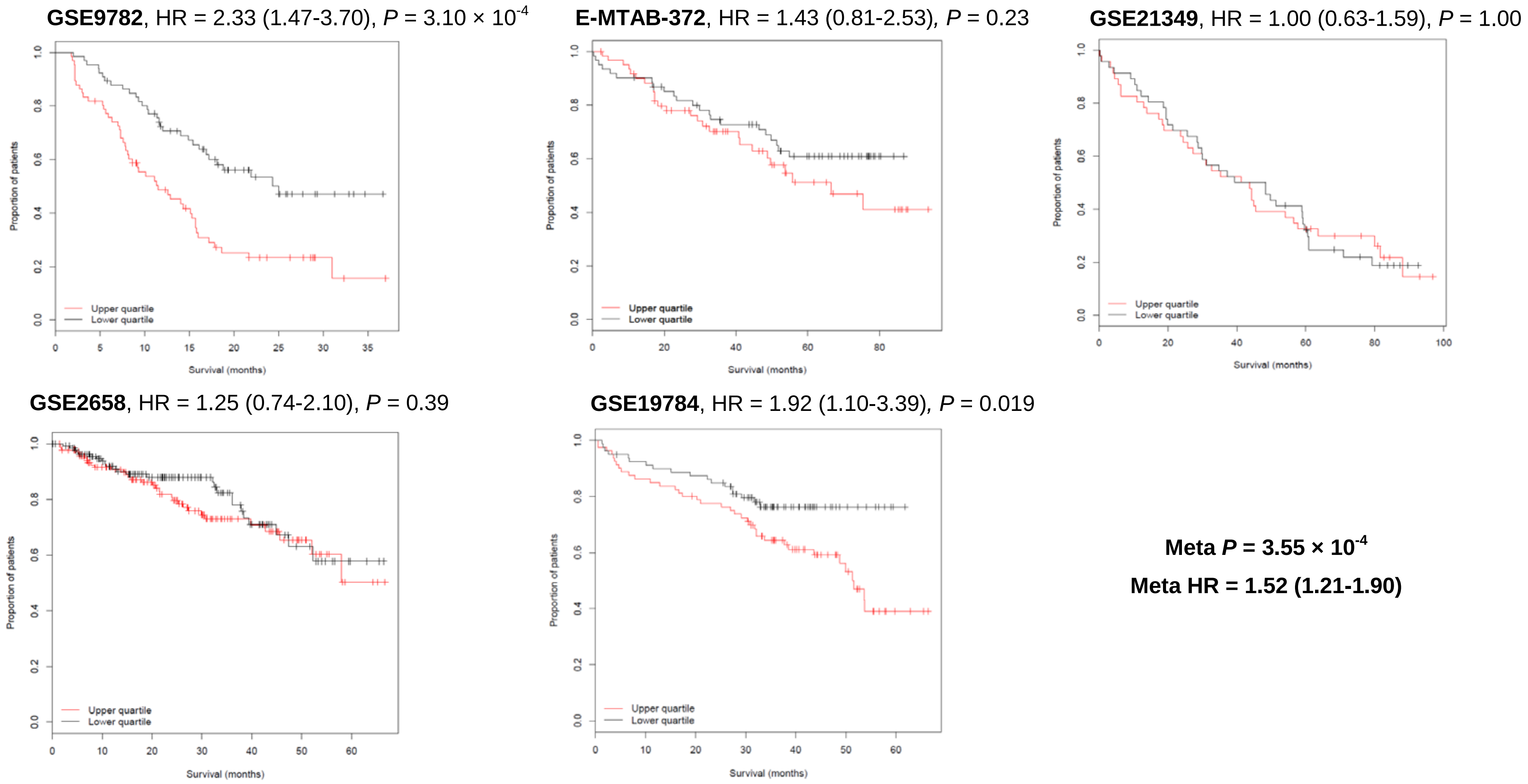

Data from: GSE9782 (n=265), GSE2658 (n=559), GSE19784 (n=320), E-MTAB-372 (n=246) and GSE21349 (n=183). Survival curves for patients with upper and lower quartile of *CDCA7L* expression shown. Vertical ticks indicate censored data points. Cox regression analysis was used to estimate expression-specific hazard ratios (HR) for each dataset with 95% confidence interval. Overall statistical significance was combined using a fixed-effects meta-analysis.

Supplementary Table 1. eQTL analysis.

SNP	Risk allele	Gene	Probeset ID	*P* value	Direction
rs4487645	G	SP4	6671_at	0.65	
		DNAH11	8701_at	0.63	
		CDCA7L	55536_at	**1.95×10^{-36}**	Increase
		RAPGEF5	9771_at	0.83	
		STEAP1B	256227_at	0.98	
		IL6	3569_at	1.00	
		TOMM7	54543_at	1.00	

The relationship between SNP rs4487645 genotype and gene expression was assessed in CD138-selected MM plasma cells using Affymetrix Human Genome U133 2.0 Plus Array data from 183 MRC Myeloma IX trial patients, 658 Heidelberg patients and 608 US patients as recently described[2,3]. Association between SNP genotype and gene expression of genes was evaluated based on the significance of linear regression coefficients. cis-meQTL analysis for all genes in the 1 Mb region spanning SNP rs4487645. Probeset ID refer to Affymetrix U133 2.0 plus array custom chip definition file (CDF v.17) mapping to Entrez genes[2,3]. *P* value < 0.05 after adjustment for multiple testing emboldened. Direction of eQTL with respective to the risk allele of rs4487645 shown for putative association.

Supplementary Table 2. Functional annotation of SNPs in linkage disequilibrium (r^2 ≥ 0.8) with rs4487645 on chr7.

SNP	Position	LD (r^2)	GERP	PhastCons	DNAse	H3K4Me1	H3K4Me3	H3K27Ac	Chromatin state	Proteins bound	RegulomeDB
rs7785157	21927082	0.92	2.60	0.00					Heterochromain; low signal		No Data
rs57104699	21928079	0.92	-1.65	0.02					Heterochromain; low signal		6
rs6948632	21929452	0.90	-0.67	0.00					Heterochromain; low signal		6
rs75341503	21936698	0.94	-0.16	0.00					Heterochromain; low signal		No Data
**rs4487645**	**21938240**	**1.00**	**-4.66**	**0.00**					**Strong enhancer**	**IRF4,PU1**	**1b**
rs56333627	21939032	0.89	-8.08	0.00					Weak enhancer		No Data
rs55714084	21939089	0.97	2.87	0.00					Weak transcribed		No Data
rs10659842	21940358	0.84	-0.07	0.00					Weak transcribed		No Data
rs7971	21940960	0.93	-3.27	0.00					Weak transcribed		6
rs56249828	21944607	0.84	-5.36	0.00					Transcriptional elongation		5

Data are shown for rs4487645 and SNPs in linkage disequilibrium (LD) (r^2 ≥ 0.8 in 1000 Genomes EUR Phase 1 data) on chr7 (human genome NCBI build 37), with histone marks (H3K4Me1, H3K4Me3, H3K27Ac), DNAse hypersensitivity sites and transcription factor binding from HaploReg (v4.1)[4] and ENCODE project data[5]. DNase hypersensitivity in grey and histone modifications in green for enhancer marks and orange for promoter marks in GM12878, with annotated chromatin states determined by the ChromHMM 15-state model[6]. Also indicated are genomic evolutionary rate profiling (GERP)[7], PhastCons[8] and RegulomeDB (v1.1)[9] scores (see http://regulome.stanford.edu/help for score annotations). Data for rs4487645 emboldened.

Supplementary Table 3. Clinical datasets used in this study. Patients in this study are of HapMap Utah residents of Western and Northern European ancestry (CEU).

Dataset accession number	Clinical trial	Sample size	Years of ascertainment	Age (Median)	Gender (% Male)	Type of MM cases	Analysis	References
GSE21349	MyIX	491	11	65	59	Newly diagnosed	Expression profiling, clinical outcome	10-12
EGAS00001001147	MyIX	463	11	65	59	Newly diagnosed	Exome sequencing	13
GSE2658	TT2/TT3	559	TT2: 8<br>TT3: 10	59	63	Newly diagnosed	Expression profiling, clinical outcome	14-17
GSE31161	TT2/TT3	1038	TT2: 8<br>TT3: 10	59	63	Newly diagnosed & Relapsed	Expression profiling, clinical outcome (Newly diagnosed patients)	Not available
GSE9782	APEX	528	1	62	58	Relapsed	Expression profiling, clinical outcome	18
GSE19784	HOVON65/ GMMG-HD4	328	6	57	60	Newly diagnosed	Expression profiling, clinical outcome	19
E-MTAB-372	GMMG-HD3/ GMMG-HD4/ GMMG-HD5	280	GMMG-HD3: 4<br>GMMG-HD4/ GMMG-HD5: 6	59	59	Newly diagnosed	Expression profiling, clinical outcome	20
E-MTAB-2299	GMMG-HD3/ GMMG-HD4/ GMMG-HD5	665	GMMG-HD3: 4<br>GMMG-HD4/ GMMG-HD5: 6	59	59	Newly diagnosed	Expression profiling, clinical outcome	3

Supplementary Table 4. Primers used in this study.

**ChIP-qPCR primers**	
rs4487645 risk-allele F	CTGAAACTTACAATTCAAGGTTTCACTTC
rs4487645 non-risk allele F	CTGAAACTTACAATTCAAGGTTTCACTTA
rs4487645 R	GGCTAGGGACAGATGAACCTCTT
Intergenic region F[21]	ATGTCAGGCCCATGAACGAT
Intergenic region R[21]	CATTCATGGAGTCCAGGCTT
**3C-qPCR primers**	
rs4487645 constant R	GTTCATCTGTCCCTAGCCTCTGTGAGC
CDCA7L Promoter F	ACAGTAGAGCATCCTGTACATGTTCTCTTCTCG
Control region 1 F	AGAATTCAAAATGGTGTACATGTTCTCTTCTCG
Control region 2 F	CCAATATGCCTTTGTACATGTTCTCTTCTCG
Control region 3 F	GAGCAATTGTAAGTGGTACATGTTCTCTTCTCG
Control region 4 F	ATGAAACAACATTAATGGTACATGTTCTCTTCTCG
Intersite control F	GTCAGGCCCATGAACGATAAAAGGG
Intersite control R	GGTAAGCAGATGATGATGAGCAGGCA
**Plasmid construct primers**	
CDCA7L amplicon F	CCTACCTGATCCCTTCTAAGTC
CDCA7L amplicon R	AGCCTCTTCATGCTATGTGGT
SDM F	GAAACTTACAATTCAAGGTTTCACTTATCTCTTAATTTTATCGAAGAGGTT
SDM R	AACCTCTTCGATAAAATTAAGAGATAAGTGAAACCTTGAATTGTAAGTTTC
Sequencing F	CCTACCTGATCCCTTCTAAGTCA
Sequencing R	ACAGGGTGTCTGAGGACCAG
**siRNA oligos**	
IRF4 siRNA Sense	CAGCUAGACUAUUGGGUAUdTdT
IRF4 siRNA Antisense	AUACCCAAUAGUCUAGCUGGG
NC siRNA Sense	UGGUUUACAUGUCGACUAAdTdT
NC siRNA Antisense	UUAGUCGACAUGUAAACCAdTdT
**qPCR primers**	
IRF4 F	AAATCCCGTACCAATGTCCC
IRF4 R	GGGGCACAAGCATAAAAGGT
CDCA7L F	GATGTCAGATCGGCATTGCT
CDCA7L R	TGAATGAGGATTCCTGTGGC
GAPDH F	GAAGGTGAAGGTCGGAGTC
GAPDH R	GAAGATGGTGATGGGATTTC

SUPPLEMENTARY REFERENCES

1. Meissner, T. *et al.* Gene expression profiling in multiple myeloma--reporting of entities, risk, and targets in clinical routine. *Clin Cancer Res* 17, 7240-7 (2011).
2. Mitchell, J.S. *et al.* Genome-wide association study identifies multiple susceptibility loci for multiple myeloma. *Nat Commun* 7, 12050 (2016).
3. Weinhold, N. *et al.* The 7p15.3 (rs4487645) association for multiple myeloma shows strong allele-specific regulation of the MYC-interacting gene CDCA7L in malignant plasma cells. *Haematologica* 100, e110-3 (2015).
4. Ward, L.D. & Kellis, M. HaploReg: a resource for exploring chromatin states, conservation, and regulatory motif alterations within sets of genetically linked variants. *Nucleic Acids Res* 40, D930-4 (2012).
5. An integrated encyclopedia of DNA elements in the human genome. *Nature* 489, 57-74 (2012).
6. Ernst, J. & Kellis, M. ChromHMM: automating chromatin-state discovery and characterization. *Nat Methods* 9, 215-6 (2012).
7. Cooper, G.M. *et al.* Distribution and intensity of constraint in mammalian genomic sequence. *Genome Res* 15, 901-13 (2005).
8. Siepel, A. *et al.* Evolutionarily conserved elements in vertebrate, insect, worm, and yeast genomes. *Genome Res* 15, 1034-50 (2005).
9. Boyle, A.P. *et al.* Annotation of functional variation in personal genomes using RegulomeDB. *Genome Res* 22, 1790-7 (2012).
10. Walker, B.A. *et al.* Aberrant global methylation patterns affect the molecular pathogenesis and prognosis of multiple myeloma. *Blood* 117, 553-62 (2011).
11. Walker, B.A. *et al.* A compendium of myeloma-associated chromosomal copy number abnormalities and their prognostic value. *Blood* 116, e56-65 (2010).
12. Walker, B.A. *et al.* Intraclonal heterogeneity and distinct molecular mechanisms characterize the development of t(4;14) and t(11;14) myeloma. *Blood* 120, 1077-86 (2012).
13. Walker, B.A. *et al.* APOBEC family mutational signatures are associated with poor prognosis translocations in multiple myeloma. *Nat Commun* 6, 6997 (2015).
14. Hanamura, I., Huang, Y., Zhan, F., Barlogie, B. & Shaughnessy, J. Prognostic value of cyclin D2 mRNA expression in newly diagnosed multiple myeloma treated with high-dose chemotherapy and tandem autologous stem cell transplantations. *Leukemia* 20, 1288-90 (2006).
15. Zhan, F. *et al.* The molecular classification of multiple myeloma. *Blood* 108, 2020-8 (2006).
16. Zhan, F. *et al.* Gene-expression signature of benign monoclonal gammopathy evident in multiple myeloma is linked to good prognosis. *Blood* 109, 1692-700 (2007).
17. Chen, L. *et al.* Identification of early growth response protein 1 (EGR-1) as a novel target for JUN-induced apoptosis in multiple myeloma. *Blood* 115, 61-70 (2010).
18. Mulligan, G. *et al.* Gene expression profiling and correlation with outcome in clinical trials of the proteasome inhibitor bortezomib. *Blood* 109, 3177-88 (2007).
19. Broyl, A. *et al.* Gene expression profiling for molecular classification of multiple myeloma in newly diagnosed patients. *Blood* 116, 2543-53 (2010).
20. Moreaux, J. *et al.* Development of gene expression-based score to predict sensitivity of multiple myeloma cells to DNA methylation inhibitors. *Mol Cancer Ther* 11, 2685-92 (2012).
21. Mendillo, M.L. *et al.* HSF1 drives a transcriptional program distinct from heat shock to support highly malignant human cancers. *Cell* 150, 549-62 (2012).
